# China’s biggest, most neglected health challenge: Non-communicable diseases

**DOI:** 10.1186/2049-9957-2-7

**Published:** 2013-04-05

**Authors:** Shenglan Tang, John Ehiri, Qian Long

**Affiliations:** 1 Duke Global Health Institute, Duke University, Durham, NC, USA; 2 Mel and Enid Zuckerman College of Public Health, University of Arizona, Tucson, AZ, USA; 3 School of Public Health and Management, Chongqing Medical University, Chongqing, PR China

**Keywords:** Non-communicable diseases, Infectious diseases, Evidence-based public health interventions, Health system reform, China

## Abstract

**Background:**

Over the past two decades, international health policies focusing on the fight against the human immunodeficiency virus/acquired immunodeficiency syndrome (HIV/AIDS), tuberculosis (TB), malaria, and those diseases that address maternal and child health problems, among others, have skewed disease control priorities in China and other Asian countries. Although these are important health problems, an epidemic of chronic, non-communicable diseases (NCDs) in China has accounted for a much greater burden of disease due to the ongoing rapid socioeconomic and demographic transition.

**Discussion:**

Although NCDs currently account for more than 80% of the overall disease burden in China, they remain very low on the nation’s disease control priorities, attracting marginal investment from central and local governments. This leaves the majority of patients with chronic conditions without effective treatment. International organizations and national governments have recognized the devastating social and economic consequences caused by NCDs in low- and middle-income countries, including China. Yet, few donor-funded projects that address NCDs have been implemented in these countries over the past decade. Due to a lack of strong support from international organizations and national governments for fighting against NCDs, affected persons in China, especially the poor and those who live in rural and less developed regions, continue to have limited access to the needed care. Costs associated with frequent health facility visits and regular treatment have become a major factor in medical impoverishment in China. This article argues that although China's ongoing health system reform would provide a unique opportunity to tackle current public health problems, it may not be sufficient to address the emerging threat of NCDs unless targeted steps are taken to assure that adequate financial and human resources are mapped for effective control and management of NCDs in the country.

**Summary:**

The Chinese government needs to develop a domestically-driven and evidence-based disease control policy and funding priorities that respond appropriately to the country’s current epidemiological transition, and rapid sociodemographic and lifestyle changes.

## Multilingual abstracts

Please see Additional file 1 for translations of the abstract into the six official working languages of the United Nations.

## Background

Over the past two decades, international health policies have predominately focused on achieving the Millennium Development Goals (MDGs), with emphasis on key infectious diseases of global relevance, namely HIV/AIDS, TB, and malaria, and the reduction of adverse maternal and child health outcomes. As a consequence, important progress has been made in improving access to services for the prevention, diagnosis, and treatment of HIV/AIDS, TB, and malaria, with over 2.5-20 million lives saved in low- and middle-income countries globally since 1995 [1-3]. Integration of infectious disease prevention and treatment into existing maternal and child health care has largely contributed to the reduction of the global maternal mortality ratio (MMR) from 400 per 100,000 live births in 1990 to 210 in 2010, a decline of 47% [4]. As well as that, there has been a drop in under-five mortality by 41%, from 87 per 1,000 live births in 1990 to 51 in 2011 [5].

China is on track to achieve almost all the MDGs. Following the past 30 years of economic reforms, life expectancy has reached 74.8 years, the maternal mortality ratio has dropped to 26/100,000, and the infant mortality rate is currently 12/1,000 [6]. It seems that these have, to some extent, helped to skew disease control priorities in China toward infectious disease control. The reality, however, is that rapid socioeconomic and demographic transitions associated with the reforms have led to a notable shift in the burden of illness that is currently dominated by an epidemic of chronic, non-communicable diseases (NCDs). This clearly calls for a reexamination of national disease control priorities and plans of action.

## Discussion

### The burden of NCDs in China

China has experienced, and continues to experience, epidemiological transition that is marked by a decline in the prevalence of many infectious diseases, and a dramatic increase in mortality and morbidity caused by NCDs [7-9]. In 2005, deaths from NCDs accounted for 80% of all deaths (total 10.3 million) and 70% of total disability-adjusted life-years lost (total 195.7 million) [10]. It is likely that the prevalence of NCDs will continue to rise given the advancing population aging, and increased exposure to health risk factors due to environment and lifestyle changes associated with rapid urbanization.

As with infectious diseases, NCDs disproportionately affect the poor more than the rich. According to the Chinese National Health Services Survey of 2008, the prevalence of NCDs was 23% in the rural low-income populations, which was higher than the average level in rural areas (17%) [11]. Often, the poor who are affected by NCDs are either not seeking care, or are seeking cheap, but inappropriate care for the condition (e.g. purchasing a quick fix from roadside drug stores) due primarily to limitations in availability of finances and other resources. In 2008, 35% of the low-income residents with NCDs in rural China who were referred for hospital treatment could not receive care because of financial difficulties (82%) [11]. These rural poor who were hospitalized were twice more likely to be self-discharged from hospital care for financial reasons than their urban counterparts [12]. Substantial direct costs of treatment and long-term care of NCDs, as well as indirect economic loss due to lost employment, disability, or premature deaths, not only pushes households into poverty, but also erodes social and economic stability. According to the World Economic Forum’s 2010 Global Risks Report [13], NCDs pose a greater threat to global economic development than fiscal crisis, natural disasters, corruption, or infectious diseases.

### Global health politics and the neglect of NCDs in China

In recent decades, prevention and control of NCDs has never been a high priority in China. This is unfortunate because compared to other middle-income countries, such as Thailand and Brazil, China has fallen behind several decades in developing and implementing effective policies to tackle these diseases. Consequently, the country now has more than 200 million patients with hypertension and more than 90 million with diabetes [11,14]. Only a small proportion of these patients are under effective treatment and control, and funds provided from central and local governments for control of NCDs have been very limited [11].

A survey conducted in 2009–2010 in three cities in northeast China found that 29% of urban residents had hypertension. While over 60% of hypertensive patients were not aware of their condition, less than one third of the patients received treatment and only 4% had their blood pressure adequately controlled [15]. A similar study undertaken in 2007 in rural areas of Shandong reported a higher prevalence of hypertension among the rural population and fewer patients under appropriate treatment [16]. Like hypertensive patients, 60% of diabetics do not know they have developed the disease, resulting in a high rate of hospital admission for acute diabetic complications [14]. These specific examples notably demonstrate the health system’s inefficiency to control and manage NCDs. A recent survey of institutional capacity for addressing NCDs revealed that less than half of the surveyed counties had specialized institutions for NCDs, and, in 2009, only 30% of county level Centers for Disease Control and Prevention (CDCs) implemented one or more interventions for NCDs, partly due to budgetary and personnel constraints [11]. When the health system does not effectively respond to the healthcare needs of the population, delayed diagnosis and treatment of NCDs will require more invasive treatments that often lead to substantial medical expenditures, worsened health outcomes, and other related social and economic consequences. In contrast, HIV/AIDS has attracted a lot of political attention and substantial financial resources in China, although the country had just over 750,000 HIV-positive patients in a population of almost 1.4 billion in 2010 [17].

Why is this so? One reason is that since the late 1990s, millions of dollars have been invested by international donors to support China’s fight against infectious diseases, particularly HIV/AIDS. These donors include the World Bank; Britain’s Department for International Development; the Australian government; The Global Fund to Fight HIV/AIDS, TB and Malaria; and other bilateral aid programs. Many of these organizations required matching funds from either their central or local governments as a precondition for support. More recently, the Bill and Melinda Gates Foundation has joined other organizations to invest US$50 million for HIV/AIDS control and US$33 million for TB and multidrug-resistant TB control. Unfortunately, the same level of support from international donors has not been seen for any other public health issue except tobacco control, which receives support from the Gates and Bloomberg Foundations.

Another reason is that overall government funding allocated to public health interventions and services had not relatively increased since the 1980s and 90s. Therefore, health facilities, including preventive health services facilities, have continued to rely heavily on revenues generated from the provision of clinical services that are profitable to the neglect of preventive services. Since the epidemic of SARS, funding for infectious diseases has increased, as the government has been under international and domestic pressures. However, the Chinese government has not allocated adequate financial resources to tackle the increasing challenge of NCDs, except for a few local governments in the most developed regions of the country.

Traditionally, China has never been known as a country with significant challenges of NCDs, and so, there is very little domestic voice for this emerging public health problem. For this reason, NCDs do not attract the same political pressures as SARS, both domestically and from the international community. Epidemiological evidence on this growing health burden in the country should provide the impetus for action both on the part of the Chinese government and the international health community. Given the established relationship between health and socioeconomic development, it is important that China recognizes the burgeoning epidemic of NCDs as a challenge to its emergence as a global super power, and consequently institute measures to address it.

The first author of this paper has been a World Health Organization (WHO) official for the past six years. He has had many chances to meet senior representatives from Western governments responsible for international health development. On numerous occasions, he has had the opportunity to ask them why international donors have allocated billions of dollars in support of HIV/AIDS and other infectious diseases in China, India, and other Asian countries when NCDs pose, by far, a greater public health challenge than HIV/AIDS. Often, these government representatives respond that it is “easier for their governments to justify the use of aid money for infectious diseases, as these diseases could more easily spread to the Western countries.” They argue that it would be difficult to support control of NCDs in low- and middle-income countries, although they clearly understand the heavier burden and the urgent need to address NCDs in these countries. Such a policy decision may also be underpinned by the theory of health economics as the control activities of infectious diseases are often seen as public goods/services, or at least merit goods/services, while NCDs are probably not. As the WHO reported recently, 80% of deaths from NCDs occur in low- and middle-income countries, up from 40% in the 1990s [18]. In our views, therefore, it is critically important and imperative for donor governments in Western countries to not only support low- and middle-income countries in the fight against infectious diseases, but to also recognize the urgent need to support these countries in controlling NCDs given their huge negative impact not only on health, but also on poverty and overall economic development [13].

International organizations and national governments agree that the rise of NCDs has devastating social and economic consequences for developing countries as well. In September 2011, the United Nations (UN) Assembly held a summit on the control of NCDs in New York [19]. This is the second time in UN Assembly history that a health-related issue was discussed. (The first was, not surprisingly, on HIV/AIDS control.) Unfortunately, this summit has not resulted in meaningful policies and actions, nor has it been backed by billions of dollars in donor support. The commitments that emerged from the summit were largely rhetorical, as pointed out by Thomas Bollyky in his recent article published in Foreign Affairs [20]. The political declaration only recognized the “epidemic proportions” of NCDs, but neither mandated specific methods, nor even argued for their adoption. In addition, funding for the prevention and control of NCDs was not determined. A recent publication by Ann Keeling of the International Diabetes Federation indicated that “bilateral donors, the World Bank, and the International Monetary Fund (IMF) remain reluctant to allocate resources for NCDs” although the enormous health and economic burden they place on low- and middle-income countries are clearly understood [21]. Highlighting the role of country governments in fighting NCDs, it has been argued that a “lack of funding should not be an excuse for [a] lack of action” [19].

### Tackling NCDs under China's ongoing health system reform

Prior to the UN summit, the World Bank published a report entitled, *Toward a healthy and harmonious life in China: Stemming the rising tide of NCDs*. The report paints an alarming situation that's facing China and proposes a comprehensive strategy for fighting NCDs. Over the past decade and as part of the 'Healthy China by 2020' project, the Chinese government has emphasized a more balanced development between urban and rural areas, across different regions, and between social and economic spheres. In 2009, the government launched a new round of health system reform focused on universal health coverage. In the following sections, we discuss some policy commitments and the remaining challenges for the prevention and control of NCDs.

In recent years, government commitments to, and investments in, health in China have translated into several major reform initiatives including, firstly, the establishment and expansion of health insurance schemes for rural residents, which is the New Cooperative Medical Scheme (NCMS); the unemployed or informally employed urban residents, which is the Urban Resident Basic Health Insurance (URBHI); and the Urban Employee Basic Health Insurance(UEBHI). In 2009, over 90% of the Chinese population was covered by one of the three health insurance schemes [22]. However, the service benefit packages offered by NCMS and URBHI are still very limited and focus on paying for inpatient care. The argument for a system that mainly covers inpatient services was that the expensive inpatient care was believed to push people into poverty. However, as they are chronic conditions, NCDs, such hypertension and diabetes, often require long periods of outpatient management. Substantial costs incurred from frequent visits for treatment of chronic diseases have become a major factor in medical impoverishment in China [23,24].

Although a majority of counties and cities reported that outpatient services for chronic diseases had been included in the service benefit packages of NCMS and URBHI in 2010 [25], a study in Shandong and Ningxia found a very low reimbursement level for outpatient care that ranged from less than 1% to 13% [26], depending on capacity for financial mobilization for the health insurance schemes. Even for inpatient care, patients still have to pay for over half of their total treatment expenses. In 2008, 26% of households with a member having a chronic disease in rural areas and in the western region experienced catastrophic health expenditures (defined as out-of-pocket payments for health care of more than 40% of a household’s non-food consumption), which were higher than in urban areas and wealthy regions [27]. The rural poverty rate, due to the payment of medical expenses, is eight times that of urban areas, and the rate of impoverishment is three times higher [27]. The service benefit packages are planned to gradually increase. The design of these schemes should consider the situation of the rising burden of NCDs with special attention to protecting the poor and the vulnerable from financial risks induced by health care.

Secondly, the central government has increased public health spending and directed its share towards the less-developed western and central regions, which have higher shares of the rural population. In 2011, the government committed to provide 25 Chinese yuan per person for basic public health services, including for management of NCDs [25]. Nevertheless, government expenditures on health between and within provinces are regressive due to the fiscal decentralization system. The less-developed central and western regions and poor rural areas that suffer a higher prevalence of NCDs and other public health threats continue to receive less public investment than the more affluent eastern coastal region and urban areas. Many sub-national governments do not recognize that investment in health and public goods are intertwined with economic development which remains a primary objective at the local level. A recent World Bank report on equity and public governance in China's health system reform found a low compliance in the use of earmarked allocation on health in poor provinces or counties and a skewed spending on urban development within provinces, leaving rural areas underfunded [28]. It pointed out that political commitment and stewardship by the central government is essential. In addition, increased public funding to sub-national governments should be companied with vertical monitoring of the equity, efficiency, and effectiveness of local health sectors and health outcomes.

It has been acknowledged that strengthened and reoriented primary health care will be a cost-effective way to detect, diagnose, treat, and manage NCD patients so as to alleviate the burden on higher level health services, and mitigate medical costs to both individuals and the health systems [29]. There is currently no systematic strategy for monitoring primary healthcare practices, such as NCD cases management, at the grassroots level. In the 1960s and 1970s, the rural barefoot doctors (now called village doctors), who had basic healthcare training, were paid for by the local collectives (e.g. communes) to primarily provide preventive services and encourage people to participate in public health campaigns. During that period, impressive health gains were witnessed in many rural areas of China, owing partly to the good performance of the barefoot doctors. Since China's health system was transformed to one that is market-oriented in the 1980s, most barefoot doctors became private practitioners while some abandoned practices for other reasons [30]. Now, the income of village doctors largely comes from the sale of drugs. Although they may receive a certain amount of government subsidy for carrying out public health activities (e.g. TB case management), the payment is generally low and many are not keen to engage in these time-consuming services that reduce their incomes from other profit services [31]. Even in cities, medical college graduates would not like to work at community level health facilities due to low salaries and a lack of an explicit career development path [32]. This may require appropriate incentives including financial compensation and career development opportunities to encourage primary healthcare practices and improved quality of care.

## Summary

The prevention and control of chronic NCDs have been viewed as “a development, and not just a health issue” [19]. The WHO has proposed a global goal of reducing annually, an additional 2% of age-specific deaths related to NCDs. Achievement of this goal will not only save millions of lives, but will also have meaningful economic benefits in the form of prolonged, productive lives and a reduction in the need for intensive medical care at great costs. Most low- and middle-income countries are now facing a dual burden of epidemics of NCDs and infectious diseases. These diseases share some common features, such as an overlapping high-risk population, long-term care needs, supportive interventions, and co-morbidities (e.g. diabetics among TB patients). Thus, multi-sectoral cooperation and coordination will be critical to tackle these emerging public health challenges in low- and middle-income countries through the implementation of responsive, pro-poor policies and innovative strategies.

Now is the time for urgent action by the Chinese government to develop domestically-driven and evidence-based disease control policies with adequate funding, without relying on foreign donors, while at the same time, continuing the fight against infectious diseases. There must be enough room in the political agenda for this multi-pronged approach for both groups of diseases to be successfully managed.

## Abbreviations

HIV/AIDS: Human immunodeficiency virus/acquired immunodeficiency syndrome; IMF: International Monetary Fund; MDGs: Millennium Development Goals; NCDs: Non-communicable diseases; NCMS: New Cooperative Medical Scheme; TB: Tuberculosis; UEBHI: Urban Employee Basic Health Insurance; URBHI: Urban Resident Basic Health Insurance; UN: United Nations; WHO: World Health Organization.

## Competing interests

The authors declare that they have no competing interests.

## Authors’ contributions

ST conceptualized the paper, ST and QL prepared the first draft, and JE participated in the drafting and editing of the paper. All authors read and approved the final manuscript.

## Supplementary Material

Additional file 1 Multilingual abstracts in the six official working languages of the United Nations. Click here for file
