# Supplementary material for: China’s biggest, most neglected health challenge: Non-communicable diseases
Source: Infect Dis Poverty. 2013 Apr 5;2:7. doi: 10.1186/2049-9957-2-7 (PMC3710110; doi:10.1186/2049-9957-2-7)
Supplement: Additional file 1 — Multilingual abstracts in the six official working languages of the United Nations. [file 2049-9957-2-7-S1.pdf]

Translation of the abstract into the six official working languages of the United Nations

اكبر التحديات الصحية المهملة في الصين: الأمراض غير المعدية

شينجلانج تانج و جون إيهيري و كيوان لونج

ملخص

نبذة تاريخية: على مدار العقد الماضيين انصب تركيز السياسات الصحية العالمية على مكافحة فيروس نقص المناعة البشرية /و مرض نقص المناعة المكتسب (HIV/AIDS) و مرض السل (TB) و الملاريا وكذلك الأمراض الخاصة بالحمل وصحة الطفل من بين أمراض أخرى مما أدى إلى تقلص أولويات السيطرة على إنتشار الأمراض في الصين و الدول الآسيوية الأخرى. وعلى الرغم من أهمية تلك المشكلات الصحية إلا أن وباء الأمراض المزمنة غير المُعدية (NCDS) قد تسببت في في عبء أكبر من الأمراض بسبب التغير السريع المستمر في الاقتصاديات الاجتماعية و التركيبة السكانية.

مناقشة: على الرغم من أن أمراض NCDs تعد مسؤولة عن 80% من إجمالي عبء الأمراض في الصين إلا أنها لا تزال في أدني قائمة أولويات الدولة في التحكم في الأمراض وتجتذب استثمارات هامشية من الحكومات المركزية والمحلية. وهذا بدروه يترك العديد من المرضى المصابين بحالات مزمنة بدون علاج ناجع. وقد أقرت المنظمات الدولية و الحكومات الوطنية بالأثر الاجتماعي المرع والتبعات الاقتصادية التي تسببها NCDs في الدول ذات الدخل المنخفض والمتوسط وتتضمن الصين. وعلى الرغم من ذلك إلا أن قلة من المانحين قاموا بتمويل المشروعات التي تعالج أمراض NCDs والتي تم تطبيقها في هذه الدول على مدار العقد الماضي. وبسبب ضعف الدعم من المنظمات الدولية و الحكومات المحلية لمكافحة أمراض NCDs فأن المصابين بتلك الأمراض في الصين وخصوصا الفقراء الذين يعيشون في المناطق النائية والأقل تطورا يعانون من القصور في الحصول على الرعاية الطبية اللازمة. بحيث أصبحت تكاليف الزيارات المتكررة للمنشآت الطبية و العلاج المنتظم عامل أساسي في الفقر الطبي في الصين. ويهدف هذا المقال إلى أن يدفع إلى أنه وعلى الرغم من الإصلاح المستمر في النظام الطبي في الصين سيقدم فرصة متميزة لمعالجة المشاكل الصحية العامة في الصين إلا أنه قد لا يكون كافيا لمعالجة تهديد أمراض NCDs التي بدأت في الظهور على السطح مالم تتخذ خطوات تهدف إلى ضمان توافر الموارد المالية والبشرية على خريطة مكافحة والتحكم في أمراض NCDs في هذا البلد.

ملخص: تحتاج الحكومة الصينية إلى تطوير سياسة تحكم في الأمراض مدفوعة بدوافع محلية ومبنية على الأدلة الظاهرة وأولويات تمويل تستجيب بطريقة ملائمة للانتقال الوبائي والتغيرات السوسيوديموجرافية و أساليب الحياة سريعة التغير.

Translated from English version into Arabic by Mohamed Gaafar, through

TRANSLATORS
WITHOUT BORDERS

中国最大的且被忽略的健康威胁：慢性非传染性疾病

Shenglan Tang, John Ehiri, and Qian Long

摘要

引言：过去二十多年，国际卫生政策重在预防和控制艾滋病，结核病，疟疾等传染性疾病，以及与这些疾病相关的妇幼健康问题。在这样的政策导向下，中国和其他亚洲国家均侧重于传染性疾病控制和妇幼健康的促进。尽管这些都是很重要的健康问题，中国正经历快速的社会经济和人口转型，面临严峻的慢性非传染性疾病的流行和与之相关的沉重疾病负担。

讨论：在中国，慢性非传染性疾病目前已占超过 80%的总的疾病负担，然而慢性病的预防和控制仍未被纳入国家疾病预防控制的优先领域，中央和地方政府对其投入较少。这导致了大多数慢病病人未得到有效的治疗。尽管国际组织和国家政府已经意识到慢性非传染性疾病对低、中收入国家，包括中国，带来的一系列社会经济的危害，但是过去 10 多年，很少有国际援助和支持的卫生项目致力于慢性非传性疾病的预防和控制在这些国家执行。由于缺少国际组织和国家政府对慢性非传性疾病的预防和控制的有力支持，中国的慢病病人，尤其是穷困人口和居住在农村和欠发达地区的病人，通常没有接受必须的卫生服务。另外，与慢性病相关的医疗花费，包括频繁的门诊和常规治疗也已成为医疗贫困的一个重要因素。目前，中国正在进行的卫生体制改革提供了一个特有的机会致力于目前的公共卫生问题。面对慢性病的威胁，需要一系列针对性的措施，以确保足够的经费和人力资源提供有效的慢病控制和管理。

小结：中国政府需要立足国情，制定以证据为基础的疾病控制政策和支助优先领域，以应对目前国家的流行病转型和快速的社会人口以及生活方式的改变。

Translated from English version into Chinese by Qian Long

Le défi sanitaire le plus important et le plus négligé en Chine : les maladies non transmissibles

Shenglan Tang, John Ehiri et Qian Long

Résumé

Contexte : Au cours des deux dernières décennies, les politiques de santé internationales se sont concentrées entre autres sur la lutte contre le virus de l’immunodéficience humaine ou le syndrome de l’immunodéficience acquise (VIH/SIDA), la tuberculose (TB), la malaria et les maladies liées aux problèmes de santé maternelle et infantile, ce qui a influencé les priorités en matière de contrôle des maladies en Chine et dans d’autres pays d’Asie. Bien que ces problèmes de santé soient importants, en Chine, l’épidémie de maladies chroniques non transmissibles (MNT) a représenté une charge de morbidité bien plus importante en raison de la rapide transition démographique et socioéconomique en cours.
Discussion : Bien que les MNT représentent actuellement plus de 80 % de la charge de morbidité globale en Chine, elles demeurent très loin sur la liste des priorités nationales en matière de lutte contre les maladies et ne bénéficient que d’un investissement marginal de la part des autorités locales et nationales. Cela signifie que la majorité des patients souffrant d'une maladie chronique sont laissés sans traitement efficace. Les organisations internationales et les gouvernements nationaux ont reconnu les conséquences sociales et économiques dévastatrices des MNT dans les pays à revenu moyen ou faible, comme la Chine. Peu de projets financés par donation ont toutefois été mis en place dans ces pays pour lutter contre les MNT au cours de la dernière décennie. En raison du manque de soutien important de la part des organisations internationales et des gouvernements nationaux à la lutte contre les MNT, les Chinois affectés, en particulier les pauvres et ceux qui vivent dans des régions rurales ou moins développées, continuent à ne disposer que d’un accès limité aux soins nécessaires. Les coûts associés aux traitements réguliers et aux visites fréquentes dans des centres de soin sont devenus un facteur majeur de l’appauvrissement médical en Chine. Cet article défend l’idée selon laquelle, bien que la réforme en cours du système de santé chinois offre une opportunité unique pour s’attaquer aux problèmes de santé publique actuels, elle pourrait ne pas être suffisante pour gérer la menace émergente des MNT, sauf si des mesures ciblées sont prises pour s’assurer que des moyens humains et financiers suffisants puissent être attribués au contrôle et à la gestion efficaces des MNT dans le pays.

Conclusion : Le gouvernement chinois doit développer une politique de contrôle des maladies et des priorités de financement fondées sur les faits et adaptées aux besoins nationaux pour répondre de façon appropriée à la transition épidémiologique actuelle du pays, ainsi qu'à la rapidité des changements sociodémographiques et à l'évolution des modes de vie.

Translated from English version into French by Anne-Laure Schneider, through

TRANSLATORS
WITHOUT BORDERS

Неконтагиозные заболевания – самая большая и наиболее игнорируемая проблема здравоохранения Китая

Шенлань Тан, Джон Эхаяри и Цянь Лун

Резюме

История вопроса: Из- за того, что последние два десятилетия международная политика в области здравоохранения была сфокусирована на борьбе с такими заболеваниями, как синдромом приобретённого иммунодефицита (ВИЧ/СПИД), туберкулёз (ТБ), малярия, болезни матери и ребёнка и другие, исказились приоритеты здравоохранения в Китае и других странах Азии. Несмотря на то, что вышеперечисленные болезни считаются важнейшими проблемами здравоохранения, всё же эпидемии хронических, неконтагиозных заболеваний (НКЗ) оказались куда большей проблемой в медицине. А послужили этому стремительные и продолжающиеся социально-экономические и демографические изменения.

Рассмотрение вопроса: Службы национального санитарно-эпидемиологического надзора, привлекая малорентабельные государственные и местные инвестиции, тем самым уделяют мало внимания проблеме НКЗ, хотя её масштабы достигли 80% от всех заболеваний в Китае. Такие действия приводят к тому, что большинство пациентов с хроническими заболеваниями остаются без надлежащего лечения. Международные организации и национальные правительства стран с низким и средним уровнем дохода (включая Китай) признали разрушающие социальные и экономические последствия, вызванные НКЗ. Всё же за последние десять лет в этих странах были запущены несколько донорских проектов, связанных с НКЗ. Из-за нехватки поддержки международных организаций и национальных правительств в борьбе с НКЗ, которые поражают население Китая, особенно бедные слои и те, которые проживают в сельской местности и менее развитых регионах, вопрос с ограниченным доступом к медицине не решается. Затраты, связанные с частым посещением медицинских учреждений и регулярным лечением, стали основным фактором обнищания системы здравоохранения в Китае. Эта статья утверждает следующее: несмотря на то, что текущая реформа в здравоохранении Китая и должна обеспечить уникальную возможность охватить имеющиеся проблемы здравоохранения, но она может оказаться не настолько эффективной, чтобы отвести возникшую угрозу НКЗ, если только не будут приняты

целенаправленные меры по обеспечению эффективного контроля и управления НКЗ в стране с помощью достаточного финансового и человеческого ресурса

Заключение: Правительство Китая должно развивать внутригосударственную и основанную на фактических данных политику санитарно-эпидемиологического контроля и политику приоритетного финансирования. А это в свою очередь должно надлежащим образом отвечать сегодняшнему эпидемиологическому росту и стремительным социально-демографическим переменам и изменениям стиля жизни населения страны.

Translated from English version into Russian by Olga van der Veen, through

TRANSLATORS
WITHOUT BORDERS

El desafío en salud más descuidado en China: Enfermedades no contagiosas

Shenglan Tang, John Ehiri, and Qian Long

Abstract

Antecedentes: En las últimas dos décadas, las políticas internacionales de salud que se enfocan en la lucha contra el Virus de Inmunodeficiencia Humana/Síndrome de Inmunodeficiencia Adquirida (VIH/SIDA), tuberculosis (TB), malaria y aquellas enfermedades que abordan problemas materno infantiles, entre otras, han desviado las prioridades de control de enfermedades en China y otros países asiáticos. Aunque estos son problemas de salud importantes, una epidemia de enfermedades crónicas no contagiosas (ENC), ha sido la razón para una carga de enfermedad mucho mayor debido a su rápida transición socioeconómica y demográfica.

Discusión: Aunque las ENC actualmente explican más del 80% de la carga de enfermedad total en China, continúan estando muy abajo en las prioridades de control de enfermedades de la nación, por lo que atraen una inversión mínima de parte de los gobiernos centrales y locales. Esta situación deja sin tratamiento efectivo a la mayoría de pacientes con condiciones crónicas. Las organizaciones internacionales y los gobiernos nacionales han reconocido las devastadoras consecuencias sociales y económicas causadas por las ENC en los países de ingresos bajos y medios, incluyendo a China. Sin embargo, se han implementado muy pocos proyectos financiados con donaciones que aborden las ENC en estos países en las últimas dos décadas. Debido a la falta de apoyo de las organizaciones internacionales y de los gobiernos nacionales para luchar contra las ENC, las personas afectadas en China, especialmente los pobres y los habitantes de áreas rurales y regiones menos desarrolladas, continúan teniendo un acceso limitado al cuidado necesario. Los costos asociados con las visitas a centros de salud y el tratamiento regular se han convertido en un factor importante de la escasez médica en China. Este artículo plantea que aunque la reforma en curso del sistema de salud de China brindaría una oportunidad única para enfrentar los problemas actuales de salud pública, podría no ser suficiente para abordar la amenaza emergente de las ENC a menos que se realicen los trámites necesarios para garantizar que se planeen los recursos humanos y financieros adecuados para el control y manejo efectivo de las ENC en el país.

Resumen: El gobierno chino debe desarrollar una política nacional de control de enfermedades basada en la evidencia y financiar las prioridades que respondan apropiadamente a la transición epidemiológica y a los rápidos cambios sociodemográficos y de estilo de vida.

Translated from English version into Spanish by Nestor Rojas, through

TRANSLATORS
WITHOUT BORDERS
